# Water-Vapor Sorption Processes in Nanoporous MgO-Al_2_O_3_ Ceramics: the PAL Spectroscopy Study

**DOI:** 10.1186/s11671-016-1352-6

**Published:** 2016-03-09

**Authors:** Halyna Klym, Adam Ingram, Oleh Shpotyuk, Ivan Hadzaman, Viacheslav Solntsev

**Affiliations:** Lviv Polytechnic National University, 12 Bandera str., 79013 Lviv, Ukraine; Physics Faculty of Opole University of Technology, 75 Ozimska str., 45370 Opole, Poland; Vlokh Institute of Physical Optics, 23 Dragoanova str., Lviv, 79005 Ukraine; Institute of Physics of Jan Dlugosz University, 13/15 al. Armii Krajowej, Czestochowa, 42201 Poland; Drohobych State Pedagogical University, I. Franko str., 24, Drohobych, 82100 Ukraine; V.E. Lashkaryov Institute of Semiconductor Physics of National Academy of Sciences of Ukraine, 41, Prospekt Nauki, 03680 Kiev, Ukraine

**Keywords:** Positron annihilation, Trapping, Positronium, Ceramics, Nanopores, Water sorption

## Abstract

The water-vapor sorption processes in nanoporous MgO-Al_2_O_3_ ceramics are studied with positron annihilation lifetime (PAL) spectroscopy employing positron trapping and positronium (Ps)-decaying modes. It is demonstrated that the longest-lived components in the four-term reconstructed PAL spectra with characteristic lifetimes near 2 and 60–70 ns can be, respectively, attributed to ortho-positronium (o-Ps) traps in nanopores with 0.3- and 1.5–1.8-nm radii. The first o-Ps decaying process includes “pick-off” annihilation in the “bubbles” of liquid water, while the second is based on o-Ps interaction with physisorbed water molecules at the walls of the pores. In addition, the water vapor modifies structural defects located at the grain boundaries in a vicinity of pores, this process being accompanied by void fragmentation during water adsorption and agglomeration during water desorption after drying.

## Background

Functional MgAl_2_O_4_ ceramics (MgO-Al_2_O_3_) with a spinel structure are known as excellent porous materials for humidity sensors [1–3]. These ceramics are thermally and chemically stable in comparison with other types of porous media possessing fast response to humidity changes [4]. An actual challenge is related to porous materials with a controllable microstructure, large specific surface area, high open porosity, optimal pore size, and distribution of free-volume entities [5, 6].

The effect of initial surface area of powdered binary oxide ingredients (MgO and Al_2_O_3_) on the structure of MgO-Al_2_O_3_ ceramics sintered at 1100–1400 °C was studied extensively in [7–10]. It was shown that the formation of this spinel-structured ceramics is substantially intensified with increase in sintering temperature and duration. In addition, the sintering temperature possessed an essential effect on the pore structure and exploitation properties of MgAl_2_O_4_ ceramics [7].

Traditionally, the microstructures of porous ceramics are studied using X-ray (electron, neutron) diffraction, electron microscopy, and different direct porosimetric methods [11–13]. However, the techniques of mercury and/or nitrogen intrusion porosimetry provide reliable information on open pores with radii over 2 nm [14], whereas physical processes in such ceramics depend on not only large open pores but closed nanopores too [15].

To gain knowledge about such fine free-volume entities and their effect on MgAl_2_O_4_ ceramics, it is reasonable to use the method of positron annihilation lifetime (PAL) spectroscopy, which is an alternative probe of structural characterization allowing the study of both closed and open pores at a nanoscale [16]. Two channels of positron annihilation were shown to be important in case of ceramics: positron trapping and ortho-positronium (o-Ps) decaying [17–19]. The latter process (“pick-off” annihilation) resulting from positronium (Ps) interaction with electron from environment (including annihilation in liquid water) is ended by emission of two γ-quanta [17]. In general, these two channels of positron annihilation are independent. However, if trapping sites will appear in a vicinity of grain boundaries neighboring with free-volume pores, these positron-Ps traps become mutually interconnected resulting in a significant complication of PAL data.

In this work, we use the PAL method developed in positron trapping and o-Ps-decaying modes to characterize MgO-Al_2_O_3_ ceramics sintered at 1100–1400 °C in different stages of water-vapor sorption and drying treatment.

## Methods

The MgO-Al_2_O_3_ ceramics were sintered at maximal temperatures (*T*_s_) of 1100, 1200, 1300, and 1400 °C for 2 h, as it was described elsewhere [7, 9, 20, 21]. In respect to X-ray diffraction measurements [7], the ceramics prepared at lower *T*_s_ = 1100–1200 °C are composed of the main spinel phase and a large amount of additional MgO and Al_2_O_3_ phases (up to 12 %), while the ceramics sintered at high *T*_s_ of 1300 and 1400 °C contain additionally only the MgO phase in the amount of 3.5 and 1.5 %, respectively.

The PAL measurements were performed with the ORTEC instrument (using ^22^Na source placed between two identical sandwiched samples) [7, 22] at 22 °C and relative humidity RH = 35 % after drying, 7 days of water exposure (water vapor in a desiccator at RH = 100 %), and further final drying in a vacuum at 120 °C for 4 h. Each PAL spectrum was collected within a 6.15-ps channel width to analyze short and intermediate PAL components. To obtain data on longest-lived PAL components, the same ceramics were studied within a channel width of 61.5 ps [15]. The collected spectra were analyzed with LT software [23]. In previous works [8–10], we used three-component fitting procedures under normal statistical treatment of PAL spectra accumulated near one million of elementary positron annihilation events. At high-statistical measurements (more than ten million counts), the best results were obtained with a four-term decomposition procedure. Such approach allows us to study nanopores of different sizes, responsible for o-Ps decaying. Each PAL spectrum was processed multiply owing to slight changes in the number of final channels, annihilation background, and time shift of the 0th channel. In such a manner, we obtained fitting parameters (positron lifetimes *τ*_1_, *τ*_2_, *τ*_3_, *τ*_4_ and corresponding unity-normalized intensities *I*_1_, *I*_2_, *I*_3_, *I*_4_), which correspond to annihilation of positrons in the samples of interest within a quite reliable error bar.

## Results and Discussion

**Fig. 1:**
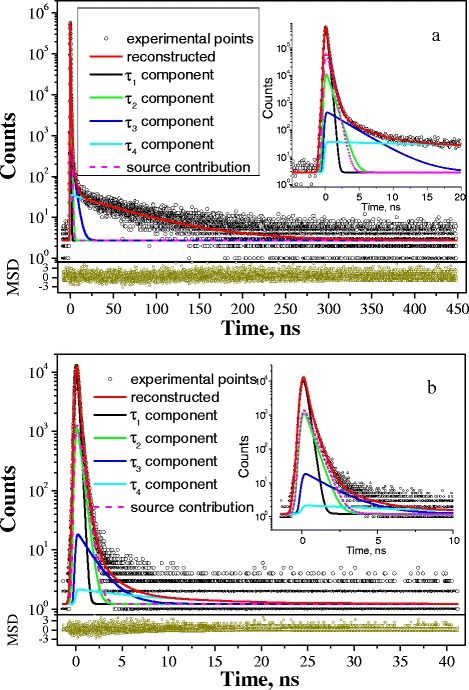
PAL spectra of MgO-Al_2_O_3_ ceramics sintered at 1400 °C registered at channel widths of 61.5 ps (**a**) and 6.15 ps (**b**), reconstructed from four-term fitting at the general background of source contribution (bottom *inset* shows statistical scatter of variance)

Typical PAL spectra of MgO-Al_2_O_3_ ceramics sintered at 1400 °C detected within 6.15- and 61.5-ps channel widths are shown in Fig. 1. Four discrete exponentially decaying components were reconstructed from these spectra using the known LT program [23]. Fitting curves for all components in the region of PAL spectra’s peaks are depicted in more detail in the inserts of Fig. 1.

As it was shown earlier [7–10], the positron annihilation in humidity-sensitive MgO-Al_2_O_3_ ceramics is revealed through two different channels related to “free” positron trapping (the intermediate component with lifetime *τ*_2_) and o-Ps decaying (two long-lived components with *τ*_3_ and *τ*_4_ lifetimes). The first component with parameters *τ*_1_ and *I*_1_ reflects mainly microstructure specificity of spinel ceramics with character octahedral and tetrahedral vacant cation sites along with input from annihilation of para-Ps atoms. The intermediate lifetime *τ*_2_ is related to the size of free-volume defects near grain boundaries, and *I*_2_ intensity reflects their amount [10]. The third and fourth components (*τ*_3_, *I*_3_) and (*τ*_4_, *I*_4_), respectively, originate from annihilation of o-Ps atoms in intrinsic nanopores of MgO-Al_2_O_3_ ceramics [7, 24].

Fitting parameters obtained within the four-component treatment of the reconstructed PAL spectra of initially dried, water-vapored, and finally dried MgO-Al_2_O_3_ ceramics sintered at 1100–1400 °C are gathered in Table 1. It is established that *τ*_1_ lifetime in the dried ceramics decreases with *T*_s_, while *I*_1_ intensity increases in respect to the amount of main spinel phase like in [7, 10]. Positrons are trapped more strongly in ceramics prepared at lower *T*_s_, as reflected in the values of the second component of the reconstructed PAL spectra. As it follows from Table 1, the numerical values of this component (*τ*_2_ and *I*_2_) decrease with *T*_s_ (so positron-trapping parameters calculated within a two-state trapping model [16] will be also changed).

**Table 1:** Fitting parameters describing PAL spectra of MgO-Al_2_O_3_ ceramics sintered at different *T*_*s*_ temperatures reconstructed from a four-term decomposition procedure

Sample	*τ*_1_ (±0.002), ns	*I*_1_ (±1), %	*τ*_2_ (±0.005), ns	*I*_2_ (±1), %	*τ*_3_ (±0.002), ns	*I*_3_ (±0.2), %	*τ*_4_ (±0.02), ns	*I*_4_ (±0.1), %
*T*_s_ = 1100 °C								
Initial drying	0.169	68	0.462	28	2.240	1.7	70.14	2.5
Water vapor	0.170	66	0.483	28	1.820	4.4	53.05	0.9
Final drying	0.172	68	0.459	29	2.215	2.1	68.29	1.9
*T*_s_ = 1200 °C								
Initial drying	0.164	73	0.443	24	2.347	1.1	70.51	2.0
Water vapor	0.160	64	0.426	31	2.047	3.8	58.67	0.4
Final drying	0.163	72	0.429	23	2.290	3.1	68.87	1.7
*T*_s_ = 1300 °C								
Initial drying	0.155	82	0.414	16	2.426	0.8	68.74	1.4
Water vapor	0.161	76	0.400	21	2.619	1.8	58.33	0.7
Final drying	0.156	82	0.421	15	2.448	0.7	68.17	1.4
*T*_s_ = 1400 °C								
Initial drying	0.152	88	0.388	11	2.504	0.7	62.32	0.8
Water vapor	0.160	77	0.409	20	2.562	2.2	57.35	0.6
Final drying	0.154	89	0.402	10	2.539	0.7	61.85	0.8

As it follows from Table 2, the calculated values of positron trapping modes in MgO-Al_2_O_3_ ceramics (average positron lifetime *τ*_av*.*_, bulk positron lifetimes in defect-free samples *τ*_b_, and positron trapping rates in defects *κ*_d_) are decreased with sintering temperature *T*_s_. These parameters are in good agreement with the amount of additional MgO and Al_2_O_3_ phases in the ceramics [7].

**Table 2:** Positron trapping modes and free-volume nanopore parameters related to o-Ps decaying determined from four-term decomposed PAL spectra of MgO-Al_2_O_3_ ceramics sintered at different *T*_*s*_ temperatures

Sample	Positron trapping modes			Free-volume parameters			
	*τ*_av_, ns	*τ*_b_, ns	*κ*_d_, ns^−1^	*R*_3_, nm	~*f*_3_, %	*R*_4_, nm	~*f*_4_, %
*T*_s_ = 1100 °C							
Initial drying	0.254	0.21	1.10	0.309	0.38	1.844	11.75
Water vapor	0.263	0.21	1.15	0.271	0.66	1.539	2.43
Final drying	0.257	0.21	1.08	0.307	0.46	1.810	8.36
*T*_s_ = 1200 °C							
Initial drying	0.232	0.19	0.94	0.319	0.26	1.852	9.62
Water vapor	0.252	0.21	1.19	0.293	0.72	1.636	1.16
Final drying	0.229	0.19	0.93	0.296	0.61	1.821	7.77
*T*_s_ = 1300 °C							
Initial drying	0.197	0.17	0.66	0.325	0.20	1.818	6.18
Water vapor	0.213	0.19	0.80	0.340	0.52	1.630	2.40
Final drying	0.198	0.17	0.63	0.327	0.20	1.807	6.06
*T*_s_ = 1400 °C							
Initial drying	0.178	0.16	0.44	0.331	0.19	1.701	3.07
Water vapor	0.211	0.18	0.78	0.335	0.63	1.613	1.74
Final drying	0.179	0.16	0.40	0.334	0.19	1.692	3.02

**Fig. 2:**
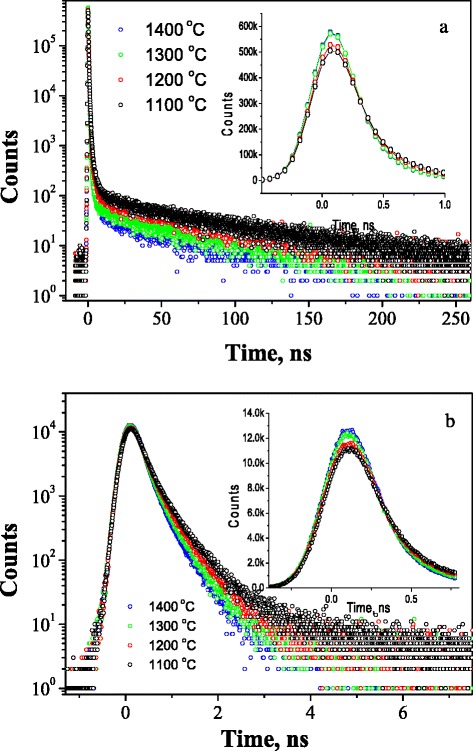
PAL spectra of MgO-Al_2_O_3_ ceramics sintered at 1100–1400 °C registered under channel widths of 61.5 ps (**a**) and 6.15 ps (**b**)

At the same time, the principal water-vapor sorption processes in the studied MgO-Al_2_O_3_ ceramics sintered at 1100–1400 °C occur to be mostly determined by o-Ps-related components in the PAL spectra reconstructed through the four-term fitting procedure (Fig. 1). Some details of PAL spectra of the ceramics sintered at different *T*_s_ are shown in the region of the main peak and long fluent decaying of coincidence counts at a certain time in Fig. 2. As it was shown earlier [7, 15], the corresponding long-lived lifetimes *τ*_3_ and *τ*_4_ reflect sizes of nanopores, and their intensities *I*_3_ and *I*_4_ are directly related to the number of these nanopores [7, 15].

So, in the initially dried MgO-Al_2_O_3_ ceramics sintered at 1100–1400 °C, the lifetime *τ*_3_ increases with *T*_s_, while intensity *I*_3_ decreases (Table 1). These changes are due to the increase in the size of small nanopores with radius *R*_3_, and their reduction is caused by increasing contact between grains. The lifetime *τ*_4_ and intensity *I*_4_ naturally decrease with *T*_s_, indicating reduction in size and number of nanopores with radius *R*_4_. The radii *R*_3_ and *R*_4_ of spherical nanopores (given in Table 2) were calculated using o-Ps-related *τ*_3_ and *τ*_4_ lifetimes in the known Tao-Eldrup model [25, 26]:

1 $$ {\tau}_{\mathrm{o}\kern0.5em \hbox{-} \kern0.5em \mathrm{P}\mathrm{s}}={\left[2\left(1-\frac{R}{R+\varDelta R}+\frac{1}{2\pi } \sin \left(\frac{2\pi R}{R+\varDelta R}\right)\right)+0.007\right]}^{-1}, $$

where Δ*R* is an empirically derived parameter (Δ*R* ≈ 0.1656 nm for polymers [18]), which describes effective thickness of the electron layer responsible for the “pick-off” annihilation of o-Ps in a hole.

**Fig. 3:**
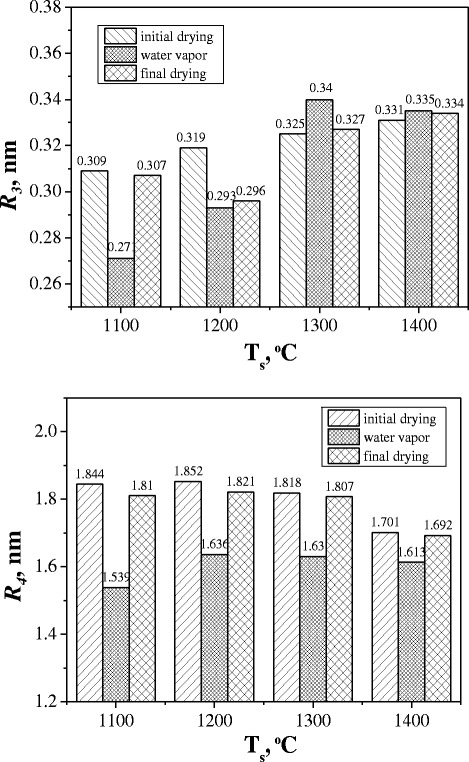
Nanopore radii *R*_3_ and *R*_4_ in MgO-Al_2_O_3_ ceramics sintered at 1100–1400 °C changed in water adsorption-desorption cycles

It is shown that the radius of nanopores *R*_3_ increases from 0.309 to 0.331 nm and *R*_4_ remains nearly at the same level (~1.8 nm) in the initially dried ceramics sintered at 1100–1400 °C (Fig. 3). In addition, free-volume fraction *f*_v_ (Table 2) was evaluated using o-Ps-related intensities *I*_o-Ps_ corresponding to the third *I*_3_ and fourth *I*_4_ components of the PAL spectra:

2 $$ {f}_{\mathrm{v}}=C\cdot {V}_f\cdot {I}_{\mathrm{o}\hbox{-} \mathrm{P}\mathrm{s}}, $$

where ${\mathit{\mathsf{V}}}_{\mathsf{f}}=\mathsf{4}/\mathsf{3}\cdot \pi \cdot {\mathit{\mathsf{R}}}_{\mathsf{o}\hbox{-} \mathsf{P}\mathsf{s}}$is the free volume of a nanopore calculated using o-Ps-related components in spherical approximation and *С* is an empirical parameter equal to 0.0018 [18].

Preferential decreasing of the lifetime *τ*_2_ in water-vapored MgO-Al_2_O_3_ ceramics and increasing of their intensity *I*_2_ demonstrates intensification of positron trapping in defects near grain boundaries filled with water. After final drying, the intensities *I*_2_ practically completely return to the initial values (character for initially dried samples), whereas *τ*_2_ lifetimes are larger in ceramics sintered at 1300 and 1400 °C. Thus, the water adsorption processes in MgO-Al_2_O_3_ ceramics are accompanied by fragmentation of positron trapping sites near grain boundaries, and, respectively, the water desorption processes are accompanied by agglomeration of free-volume voids.

Water-vapor sorption processes in the studied ceramics result in essential evolution of the third and fourth o-Ps-related components. The intensity *I*_3_ increases in all initially dried samples after water-vapor exposure, thus confirming o-Ps annihilation in water-filled nanopores through a “bubble” mechanism (with corresponding o-Ps lifetime close to 1.8 ns) [27–29]. At the same time, the lifetime *τ*_3_ decreases in more defective ceramics sintered at 1100 and 1200 °C but increases in more perfect ceramics sintered at 1300 and 1400 °C. After final drying, the intensity *I*_3_ for ceramics sintered at 1100 and 1200 °C is not returned to initial values. This confirms the remainder of sorbed water in the nanopores with size near 0.3 nm and slight desorption ability of these MgO-Al_2_O_3_ ceramics samples (Fig. 3). In MgO-Al_2_O_3_ ceramics sintered at 1300 and 1400 °C, the intensity of the third component returns to initial value, confirming high efficiency of water adsorption-desorption processes.

Another mechanism of water-vapor sorption processes similar to one reported in [30] is realized in the studied MgO-Al_2_O_3_ ceramics through the fourth component of the PAL spectra. Unlike the third component, the intensity *I*_4_ decreases in water-vapor exposure ceramics samples. Since this intensity does not drop to zero being within 0.4–0.9 % domain, it should be assumed that there exists a fraction of closed nanopores where o-Ps are trapped [15]. After final drying (in a vacuum at 120 °C for 4 h) of the ceramics samples previously exposed to water vapor, the initial pore size tends to be restored (Table 2 and Fig. 3). However, it does not recover entirely, suggesting that some fraction of water molecules remain adsorbed. The intensity *I*_4_ does not return to initial value in ceramics sintered at 1100 and 1200 °C with poorly developed open porosity (Table 1). Most probably, physically adsorbed water is not fully eliminated at 120 °C in these ceramics samples. The decreased *τ*_4_ value for ceramics dried after water-vapor exposure can be connected with formation of thin layers of water molecules covering the walls of pores with radii of 1.5–1.8 nm, which are not completely removed after vacuum annealing at 120 °C for 4 h.

## Conclusions

The method of PAL spectroscopy in high-measurement statistics is employed to study water-vapor sorption processes in MgO-Al_2_O_3_ ceramics sintered at 1100–1400 °C temperatures for 2 h. It is shown that positrons are trapped more strongly in the ceramics obtained at lower *T*_s_, which was reflected in the second component of the four-term decomposed PAL spectra. The third and fourth longest-lived components in these spectra are due to annihilation of o-Ps atoms in the nanopores, the corresponding radii being calculated from *τ*_3_ and *τ*_4_ lifetimes using the known Tao-Eldrup model. The final drying in a vacuum at 120 °C for ceramics previously exposed to water vapor does not restore initial pore size, confirming sensitivity of PAL method to the amount of water molecules adsorbed in the nanopores. The Ps annihilation in nanopores with adsorbed water vapor is shown to occur via two mechanisms: (1) o-Ps decaying in nanopores with radius of 0.3 nm including “pick-off” annihilation in the “bubbles” of liquid water and (2) o-Ps trapping in free volume of nanopores (1.5–1.8 nm) with physisorbed water molecules at the pore walls. The water vapor modifies defects in ceramics located near grain boundaries, this process accompanied by void fragmentation at water adsorption with further void agglomeration at water desorption after drying.
